# Gene-centered metagenome analysis of Vulcano Island soil (Aeolian archipelago, Italy) reveals diverse microbial key players in methane, hydrogen and sulfur cycles

**DOI:** 10.1007/s10482-024-01995-5

**Published:** 2024-07-02

**Authors:** Federica Angius, Geert Cremers, Jeroen Frank, Caitlyn Witkowski, Arjan Pol, Theo A. van Alen, Mike S. M. Jetten, Huub J. M. Op den Camp, Tom Berben

**Affiliations:** 1 Department of Microbiology, Radboud Institute for Biological and Environmental Sciences, Faculty of Science, Radboud University, Heyendaalseweg 135, 6525 AJ Nijmegen, The Netherlands; 2 Department of Marine Microbiology and Biogeochemistry, NIOZ, P.O. Box 59, 1790 AB Den Burg, Texel, The Netherlands; 3 School of Earth Sciences, Wills Memorial Building, University of Bristol, Queens Road, Clifton, BS8 1RJ UK

**Keywords:** Volcanic soil, Hydrogen sulfide, Metagenome, Metabolic reconstruction

## Abstract

The Aeolian archipelago is known worldwide for its volcanic activity and hydrothermal emissions, of mainly carbon dioxide and hydrogen sulfide. Hydrogen, methane, and carbon monoxide are minor components of these emissions which together can feed large quantities of bacteria and archaea that do contribute to the removal of these notorious greenhouse gases. Here we analyzed the metagenome of samples taken from the Levante bay on Vulcano Island, Italy. Using a gene-centric approach, the hydrothermal vent community appeared to be dominated by *Proteobacteria*, and *Sulfurimonas* was the most abundant genus. Metabolic reconstructions highlight a prominent role of formaldehyde oxidation and the reverse TCA cycle in carbon fixation. [NiFe]-hydrogenases seemed to constitute the preferred strategy to oxidize H_2_, indicating that besides H_2_S, H_2_ could be an essential electron donor in this system. Moreover, the sulfur cycle analysis showed a high abundance and diversity of sulfate reduction genes underpinning the H_2_S production. This study covers the diversity and metabolic potential of the microbial soil community in Levante bay and adds to our understanding of the biogeochemistry of volcanic ecosystems.

**Supplementary Information:**

The online version contains supplementary material available at 10.1007/s10482-024-01995-5.

## Introduction

Microbial life is incredibly resilient: it dwells in hot springs and calcinated deserts, in acid pools and polar ice, at kilometers altitude in the air and under the seabed, and as more recently discovered, in the deepest rocks of the planet (Abe and Horikoshi 2001; D’Amico et al. 2006; Holden et al. 1998; Huang et al. 2013; Li et al. 2020; Ma et al. 2010; Rainey and Oren 2006). The microbes found in these extreme habitats are called extremophiles. They are called “extreme” because they thrive in inhospitable regions for human beings. However, these microbes have evolved a set of adaptations making extreme environments their preferred niche.

Vulcano Island is one of the best-studied ‘extreme’ sites, yielding more genera of hyperthermophiles than any other hydrothermal environment (Amend et al. 2003). Vulcano Island is part of the Aeolian archipelago in the southern Tyrrhenian Sea. Each island in the Aeolian arc has a different tectonic and magmatic history, and their activities are not always correlated in time (Crisci et al. 1991). Although, since the last eruption in 1888–1890, the principal crater on Vulcano Island “La Fossa” has been in a quiescent state, high-temperature fumaroles and diffuse emissions of carbon dioxide (CO_2_)-rich gases still characterize the site. Furthermore, significant concentrations of hydrochloric acid (HCl), sulfur dioxide (SO_2_), hydrogen sulfide (H_2_S), hydrofluoric acid (HF), carbon monoxide (CO), methane (CH_4_), and hydrogen (H_2_) were also reported (Amend et al. 2003).

Levante bay represents the get-together point where CO_2_-rich gases, coming from high-temperature fumaroles concentrated near the active edifice of the La Fossa crater, merge. A recent analysis on Levante bay soil showed that temperatures ranged from 30.9 to 37.5 °C and soil CO_2_ fluxes were strongly fluctuating (Fagorzi et al. 2019). The gas composition at different sites was never the same. Mostly CO_2_ and N_2_ were the dominant gases, while H_2_S and H_2_ were only found in specific spots. Moreover, alkanes dominated the organic fraction of gases, mainly represented by ethane and propane (Fagorzi et al. 2019).

Especially at Levante bay, the temperature and composition of the hydrothermal fluids are difficult to interprete due to the highly variable chemical composition of fluids from various sampling sites. These variations are mainly responsible for the often-considerable differences in chemical energy released by microbially catalyzed redox processes, and thus may account for the high biological diversity of thermophiles observed on Vulcano Island (Amend et al. 2003).

Three of the most well-characterized and studied acidophiles were isolated from Vulcano Island (Huber and Stetter 1989; Simmons and Norris 2002). They belong to the *Acidihalobacter* genus, a Gram-negative, halophilic, iron-and sulfur-oxidizing, chemolithoautotrophic, extreme acidophiles (Khaleque et al. 2019). Moreover, researchers isolated the thermophilic *Acidianus infernus* and representatives of the genus *Thermoplasma* from the hydrothermal vents of Vulcano (Simmons and Norris 2002).

In a metagenomic study of submarine samples, Antranikian and colleagues describe its microbial population composed by Proteobacteria*,* Thermoprotei, and Thermococci (Antranikian et al. 2017). A more recent study on 16S rRNA gene amplicons of Levante bay identified as the most dominant phyla Actinobacteria, Proteobacteria, and Firmicutes (Fagorzi et al. 2019).

The aim of our research was to use a gene-centric approach to study the chemolitho(auto)trophic microbial community and their metabolism of the available gaseous electron donors. Although many attempts have been made to study the physiological, phylogenetic, and genomic diversity at Vulcano, very little is known about the in situ metabolic strategies used by the microbial community at this site. Therefore, we investigated the diversity of bacterial communities and uncovered the major metabolic pathway that shapes the Levante bay ecosystem. This will improve our understanding of the microbial dynamics in volcanic ecosystems.

## Materials and methods

### Sampling

Samples were collected by Caitlyn Witkowski (NIOZ institute, Texel, NL) in May–June 2017 at Vulcano Island, Sicily, Italy. Surface soil samples were taken from two areas of Levante bay in Vulcano Island Punto 1 (38°25′2″N; 14°57′34″E) and Punto 7 (38°25′7″N; 14°57′34″) (Amend et al. 2003). Both sites were sampled five times. From Punto 1, samples were gathered in a straight line with 2 m between each sampling spot; while from Punto 7, samples were acquired in the dry land, next to the water, and in the water. Soil samples were stored at 4° C in sterile 50 ml tubes and after transfer to the lab (within 7 days) frozen at − 20° C until used.

### Nucleic acid extraction

For metagenomic analysis, nucleic acid was extracted from soil samples using two kits and three detergent-based methods. PowerSoil DNA Isolation Kit (MO BIO Laboratories Inc., Carlsbad, CA, USA) and FastDNA™ SPIN Kit for Soil (MP Biomedicals, Santa Ana, CA, USA) were performed according to the manufacturer’s instructions. Cetyltrimethylammoniumbromide (CTAB)-based extraction (Zhou et al. 1996), dithiothreitol (DTT)-based extraction (Bürgmann et al. 2001), and polyethylene glycol (PEG)6000-based extraction (Lever et al. 2015) are described below.

### CTAB-based extraction

Soil samples (250 mg) were incubated with 675 µl of CTAB buffer (100 mM Tris, 100 mM EDTA, 100 mM Na_2_HPO_4_, 1.5 M NaCl and 1% CTAB at pH 8.0), 50 µl lysozyme (10 mg/ml, 66,200 U/mg), 30 µl RNase A (10 mg/ml) for 30 min at 37 °C. Then, 50 µl of proteinase K (10 mg/ml, 20 U/mg) was added to the samples and incubated for 30 min at 37 °C. The mixtures were further incubated with 150 µl of 10% SDS for 2 h at 65 °C. One volume of phenol/chloroform/isoamylalcohol (25:24:1) was used to extract DNA. After 20 min at 65 °C, the upper water phases obtained following centrifugation were added to one volume of chloroform/isoamylalcohol to remove phenol traces. This time the upper water phases were added to 0.6 volume of isopropanol to achieve DNA precipitation. The pellets concentrated after centrifugation were washed with 70% ethanol and centrifuged for 10 min. Air-dried pellets were resuspended in 20 µl of diethylpyrocarbonate (DEPC) water.

### DTT-based extraction

Soil samples (800 mg) were added to 1 ml extraction buffer (50 mM NaCl, 50 mM Tris–HCl, 50 mM EDTA, and 5% SDS at pH 8.0), 500 µl glass beads, and 1 µl of 1 M DTT. Samples were shaken with a bead-beater for 1 min and subsequently centrifuged for 3 min. The nucleic acids were purified from the supernatant using 0.5 volume of both phenol and chloroform/isoamylalcohol. After centrifugation, the upper water phases were transferred to a new tube and added to an equal chloroform volume. DNAs were precipitated by adding 0.1 volumes of 3 M sodium acetate solution and 0.7 isopropanol to the water phases and upon 30 min centrifugation. The pellets were washed with 500 ml of 70% ethanol. Air-dried pellets were resuspended in 20 µl of DEPC water.

### PEG6000-based extraction

Soil samples (500 mg) were added to 750 µl of 120 mM phosphate buffer and 2 ml glass beads. Samples were bead-beaten for 1 min and then incubated with 30 µl of RNase for 15 min at 37 °C. Further lysis was achieved by adding 200 µl of SDS 10% and setting at 80 °C for about 15 min. After centrifugation, 450 µl of 1.6 M NaCl/PEG600 were added to the supernatant and incubated at room temperature for 30 min. The pellets obtained after centrifugation were resuspended in 500 µl sterile Milli-Q water. 200 µl of 75 M ammonium acetate were added, and samples were incubated at − 20 °C for 5 min to precipitate DNA. The pellets were washed with one volume of absolute ice-cold ethanol. The air-dried pellet was resuspended in 20 µl of DEPC water.

The DNA was quantified using a Qubit Fluorometer (Thermo Fisher Scientific, Waltham, MA) while its quality was assessed by UV–vis spectroscopy (Nanodrop, ND-1000, Isogen Life Science, The Netherlands).

### Metagenome sequencing and analysis

When available, DNA (1 ng) from two extraction methods was used for MiSeq library preparation. The genomic DNAs were fragmented and adapters ligated in the same step using the Illumina Nextera® XT Library Prep Kit according to the manufacturer’s instructions (Illumina, San Diego, USA). The libraries were normalized to 4 nM, and sequencing was performed with an Illumina MiSeq (Illumina, San Diego, USA) using the 300 paired-end sequencing protocol.

Quality-trimming, adapter removal, and contaminant-filtering of Illumina paired-end sequencing reads were performed using BBDUK (BBTOOLS version 37.17). Trimmed reads for all samples were co-assembled using metaSPAdes v3.10.01 (Nurk et al. 2017) at default settings. MetaSPAdes iteratively assembled the metagenome using kmer sizes 21, 33, 55, 77, 99, and 127. Reads were mapped back to the metagenome for each sample separately using Burrows-Wheeler Aligner 0.7.152 (BWA) (Li and Durbin 2010), employing the “mem” algorithm. The generated sequence mapping files were handled and converted as needed using SAMtools 2.13 (Li et al. 2009). Metagenome binning was performed for contigs longer than 2,000 bp employing four different binning algorithms: COCACOLA (Lu et al. 2017), CONCOCT (Alneberg et al. 2014), MaxBin 2.0 2.2.3 (Wu et al. 2014) and MetaBAT 2 2.10.2 (Kang et al. 2015). The resulting bin set was supplied to DAS Tool 1.09 (Sieber et al. 2018) for consensus binning to obtain the final optimized metagenome assembled genomes (MAGs). The quality of the MAGs bins was assessed through single-copy marker gene analysis using CheckM 1.0.710 (Parks et al. 2015).

The MAGs were annotated using Prokka (version 1.10) (Seemann 2014), and were visualized and manually checked using Artemis and Blast searching (Rutherford et al. 2000). 16S rRNA gene-based taxonomy was performed by mapping the metagenome sequence reads to the SILVA reference database (release 132) (Quast et al. 2013).

### Metabolic reconstruction

The metabolic potential of all MAGs and the unbinned contigs was determined using an in-house pipeline, as explained in (Picone et al. 2020).

### *Iron*-related genes analysis

The iron-related genes of the community were analyzed using FeGenie, a bioinformatics tool that uses a comprehensive database of hidden Markov models (HMMs) based on genes related to the iron acquisition, storage, and reduction/oxidation in Bacteria and Archaea (Garber et al. 2020).

### Data visualization

Figures were generated using ggplot2 (Wickham 2016) and plotly (Sievert 2020) with Rstudio (RStudio, 2020).

## Results and discussion

To identify Levante Bay’s soil microbial community, we collected samples from Punto 1 (P1) and Punto 7 (P7). From a total of 10 samples, only 4 of them (Punto 7—A, Punto 7—B, Punto 7—C, and Punto 7—in water) yielded high-quality DNA, despite using five different DNA extraction methods. DNA extraction is especially difficult for acidic soils containing clay—like the one on Vulcano Island—given the tight binding of DNA strands to clay soil humic particles. Additionally, extracellular DNA binds to soil humic substances and these are co-purified resulting in inhibition of enzymes such as restriction endonucleases and DNA polymerase (Högfors-Rönnholm et al. 2018).

The Illumina sequencing yielded 19,465,333 reads evenly distributed across the samples. Reads were pooled and analyzed together; 74% were trimmed quality-controlled metagenome reads, 92% were mapped to contigs. The metaSPAdes assembly resulted in 77,835 contigs binned into 142 Metagenome Assembled Genomes (MAGs); from the MAGs, 29 were more than 90% complete (Table S2).

### Community analysis

We studied the bacterial community using a top-down approach. We started with a 16S rRNA gene analysis, and we continued with an analysis of genes representing significant metabolic pathways. Then, we correlated all the information with the assembled MAGs (see Supplementary Table S1 for an overview).

Using the Silva 16S rRNA gene database (Quast et al. 2013) we could classify 254 genera belonging to 22 phyla—which is similar to recent work where 23 phyla were recovered from an amplicon analysis on samples of Levante Bay (Fagorzi et al. 2019). The latter study focusing on a variable region of the 16S rRNA gene with high throughput got more (rare) biodiversity on the genus level. They could classify 520 bacterial genera.

**Fig. 1:**
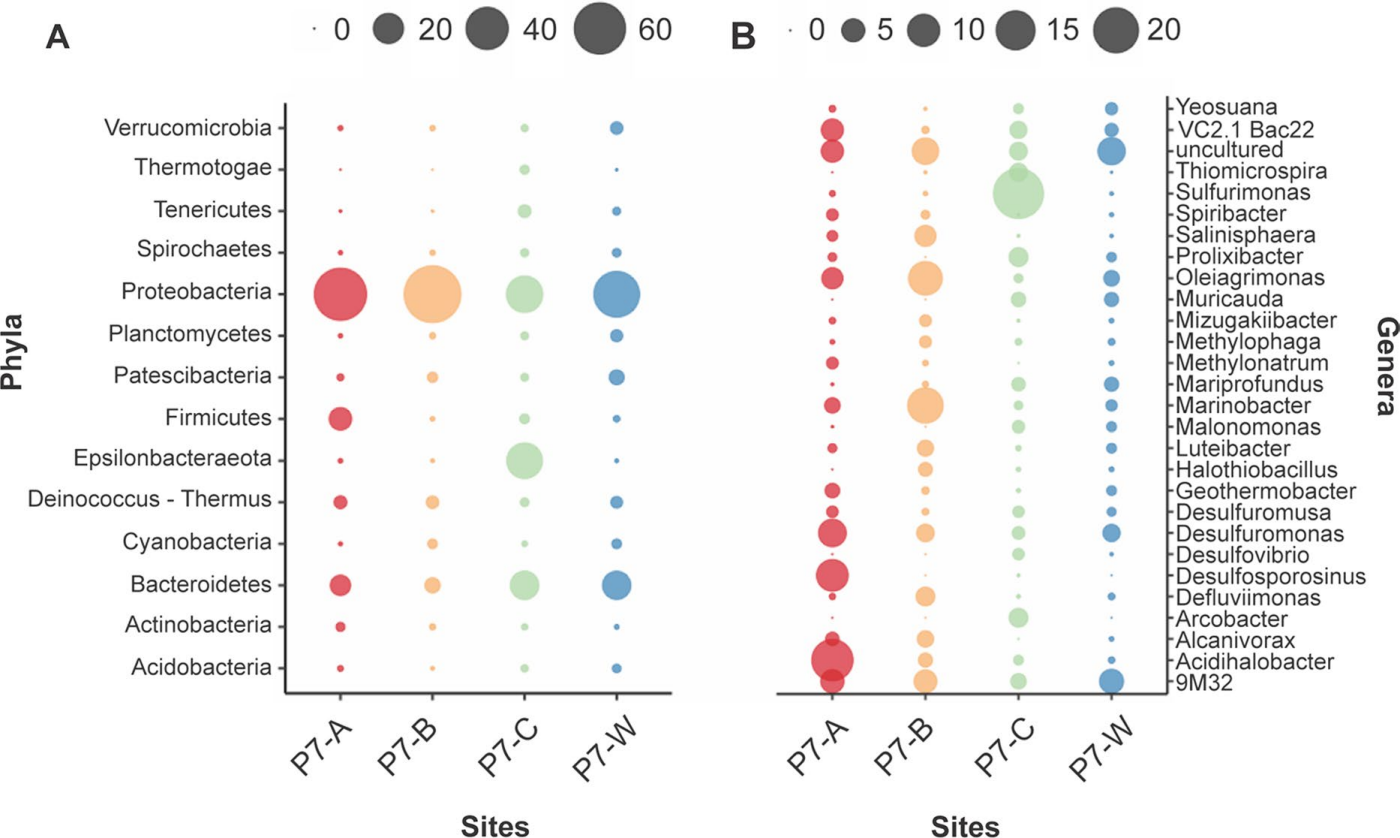
Microbial community relative abundance of phyla and genera. **A** Phyla with a relative abundance equal to or higher than 1% are shown. **B** Genera of the four most abundant phyla (Proteobacteria, Bacteroidetes, Epsilonbacteraeota, and Firmicutes) with a relative abundance equal to or higher than 1% are shown. Different colors indicate different samples: Punto 7-A (P7-A), Punto 7-B (P7-B), Punto 7-C (P7-C), and Punto 7-in water (P7-W). For clarity, the data shown here are related to FastDNA extraction. Results from other extractions are given in Table S1

The P7 sample was strongly dominated by *Bacteria*—with an average contribution of 88%—represented by Proteobacteria, Bacteroidetes, Epsilonbacteraeota, and Firmicutes (Fig. 1A). Hydrothermal vents are well-known reservoirs of diverse environmental Proteobacteria. The findings are in line with previous observations in which a high abundance of Proteobacteria was detected in hydrothermal vent fluids, vent-associated sub-surfaces as well as in the surrounding sea-water (Antranikian et al. 2017; Campbell et al. 2006; Fagorzi et al. 2019; Longnecker and Reysenbach 2001; Moyer et al. 1995; Nakagawa et al. 2005). Picone et al. (2020) described a different composition of the bacterial community retrieved from geothermal soil sampled from the Favara Grande nature reserve on the volcanic island of Pantelleria, 100 km south-west of Sicily and 70 km north-east of Tunisia. They identified Chloroflexi as the most common phyla in the top-soil, followed by Actinobacteria, Firmicutes, and Verrucomicrobia (Picone et al. 2020). The altered community structure is most likely caused by the much lower H_2_S concentrations in the geothermal emissions at Pantelleria (Duchi et al. 1994; Diliberto et al. 2021).

The proteobacterial genera that were most represented in the Levante Bay are *Sulfurimonas* (24.9%), *Acidihalobacter* (*16.9%*), *Marinobacter* (12.3%), and *Oleiagrimonas* (11%), all four are mesophilic and widespread in hydrothermal ecosystems (Fig. 1B). Species from these genera are often used in bioleaching processes (Sajjad et al. 2019). *Sulfurimonas* species (phylum Epsilonbacteraeota; Waite et al. 2017) are commonly isolated from marine environments (Han and Perner 2015). It seems that multiple copies of the enzyme sulfide:quinone reductases and hydrogenases may play a pivotal role in its flexibility to colonize various environments (Han and Perner 2015). Consistently, inhabitants of Vulcano island soil are the acidophilic and halotolerant species of the *Acidihalobacter* genus. This genus consists so far of four members, three of which—*A.* *prosperus* DSM 5130, *A. prosperus* DSM 14174, and *A. ferrooxidans* DSM 14175—were isolated from Porto di Levante and hydrothermal pools on Vulcano island, respectively. Their unique feature to simultaneously tolerate acid and saline stress makes them a potential candidate to bioleach ores with brackish or saline process waters under acidic conditions (Khaleque et al. 2019). Ferrous iron and reduced sulfur compounds are used as electron donors of *Acidihalobacter* species. The *Marinobacter* genus comprises widespread marine bacteria found in localities as diverse as the deep ocean, coastal seawater and sediment, and hydrothermal settings (Handley and Lloyd 2013). Species of this genus can contribute to the degradation of hydrocarbons in oil-polluted sediment and can oxidize inorganic compounds, such as reduced iron, arsenic and manganese ions (Handley and Lloyd 2013). The *Oleiagrimonas* genus represents two species isolated from tidal flat sediments in South Korea and China. *Oleiagrimonas soli* was isolated from oil-contaminated saline soil, suggesting the strain might play a unique role in removal of long chain hydrocarbons (Yang et al. 2017).

The most abundant Firmicutes genus was *Desulfosporosinus* (9.8%). Its members have often been found in acidic mine environments, indicating that it could play a crucial role as a sulfate-reducer within acidic environments (Mardanov et al. 2016).

The most abundant Bacteroidetes genus was most closely related to the *VC2.1 Bac22* sequence group (4.6%), for which no cultured representatives are known. Sequences of this group are dominant representatives on the bio-anode in a biowaste treating bioelectrochemical systems and commonly found in anaerobic digestion systems (Bridier et al. 2015).

The archaeal community (12% of total 16S rRNA abundance) exhibits also lower biodiversity than the Bacteria, and is represented by Nanoarchaeota, which reaches a relative abundance of 4% in P7-W, and Euryarchaeota in lower quantity (Table S1). The sequences assigned to the Nanoarchaeota phylum were all affiliated to Woesearchaeia, often found in marine environments with high organic matter content. Their genomes are small with limited metabolic capabilities. For this reason, they show a symbiotic or parasitic lifestyle (Gründger et al. 2019; Wang et al. 2019). The Euryarchaeota phylum was represented by members of the classes Halobacteria, Thermoplasmata, and Methanomicrobia.

It is worth noting that reads from only two extraction methods—PowerSoil kit and PEG6000—were mapped against Asgardaeota*,* particularly Heimdallarchaeota (Table S1). Heimdallarchaeota is the most probable candidate for the archaeal protoeukaryote. While Loki- and Thorarchaeia were associated with anaerobic lifestyle, oxygen-dependent metabolic pathways have been found in Heimdallarchaeota members (Russum et al. 2021).

We further investigated the capacity of the microbial players to utilize the gases present at Levante Bay, searching for the presence of essential genes of central metabolic pathways in the assembled contigs by HMM profiling.

### Metabolic potential of the microbial population of Vulcano soil

The dynamic fluctuations in concentration of (gaseous) electron donors and acceptors are likely responsible for the microbial diversity in Levante Bay. Amend et al. (2003) reported high concentrations of CH_4_ and CO_2_ and moderate concentrations of SO_4_^2−^, H_2_ and CO. In a recent article, Fagorzi et al. (2017) reported that—in the same year and month of the sampling of our study—CO_2_ fluxes were still intense and N_2_ was the dominant gas component. The concentration of other gases, such as H_2_S, H_2,_ and CH_4,_ were vastly different among selected sites (Fagorzi et al. 2019).

Despite the large number of hyperthermophilic genera cultivated from Vulcano soil, very little is known about the relation between gases and metabolic strategies used by the microorganisms. By analyzing functional genes of the methane, hydrogen and sulfur cycles by HMM profiling (Eddy 2011), expressed as a percentage of genes in the entire metagenome and its projection on most complete and low redundant MAGs, we provide more details on the microbial metabolic potential of Vulcanic soil.

### Methane oxidation and methanogenesis

**Fig. 2:**
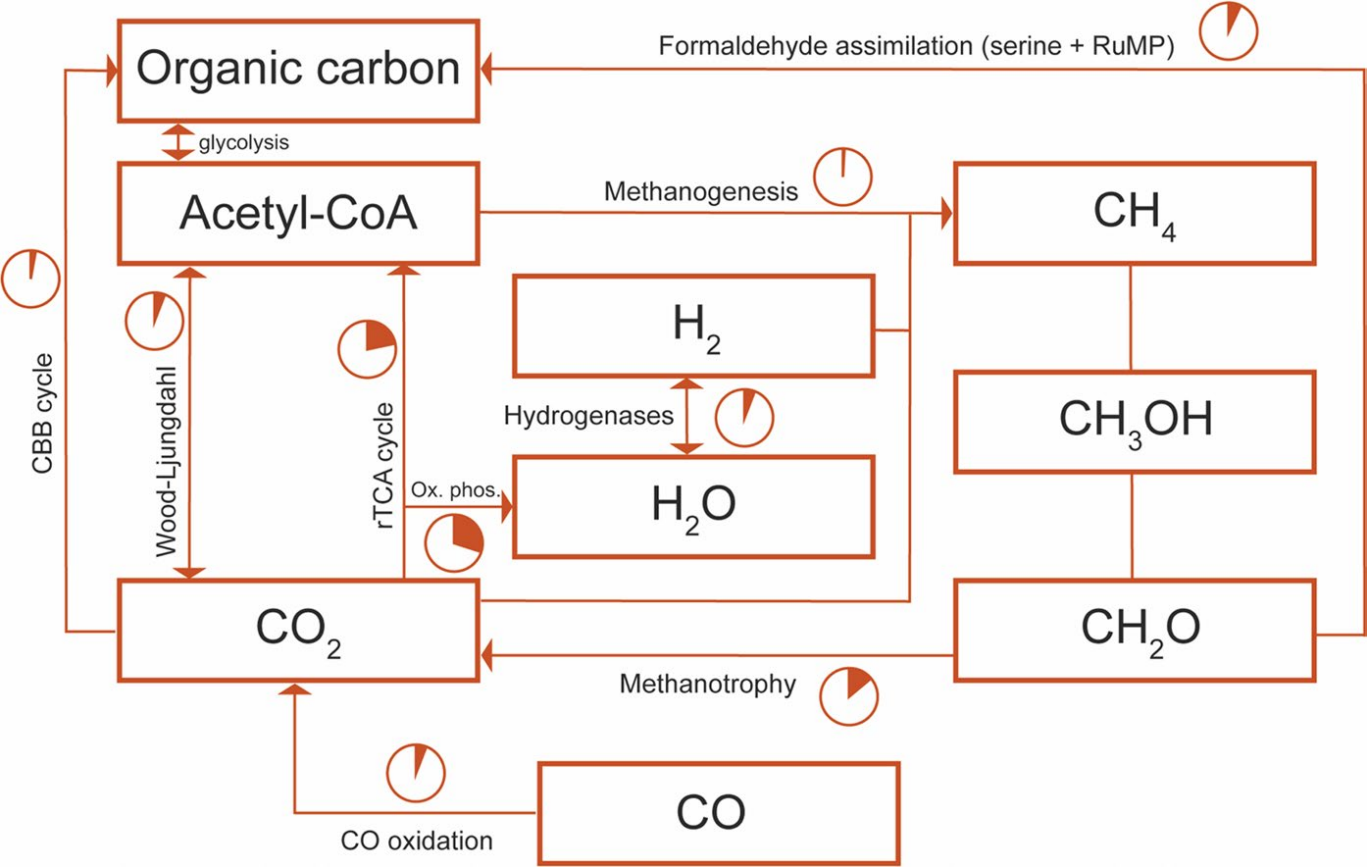
Carbon cycle overview of Vulcano metagenome. The relative abundance of genes involved in different pathways is calculated as a proportion of the total amount of genes involved in the carbon cycle

Atmospheric CH_4_ concentrations are available in soil, where methanotrophic bacteria reside. We can recognize two different types of methane-oxidizing bacteria. Low-affinity methanotrophic bacteria grow in areas of high CH_4_ concentration, such as saturated soils in wetlands and other moist environments. While, in regions with low CH_4_ concentrations, high-affinity methanotrophic bacteria make use of the CH_4_ in the atmosphere to grow, rather than relying on CH_4_ in their immediate environment (Cai et al. 2016). Methane oxidation allows methanotrophic bacteria to use methane as a source of energy. In the first step—catalyzed by the particulate methane monooxygenase (pMMO)—CH_4_ is converted into methanol (Fig. 2). One complete *pmo* operon (*pmoCAB*) 39% similar to the one present in *Methylobacter luteus* was found on a 3157 kb contig in our dataset. We did not retrieve single or partial genes encoding PmoA, PmoB, PmoC, or sMMO from the contigs.

The second step proceeds with the conversion of methanol to formaldehyde by pyrroloquinoline quinone-dependent methanol dehydrogenases (PQQ-MDH) (Fig. 2). These enzymes comprise not only the calcium-dependent MxaFI type, but also the recently discovered lanthanide-dependent XoxF-type (Pol et al. 2014; Keltjens et al. 2014). Since then, the XoxF-type PQQ-MDH has been found in many other environments and microbes (Ochsner et al. 2019; Picone et al. 2020; Schmitz et al. 2021; Smith et al. 2018). From our metagenome contigs, 30 PQQ-MDHs were retrieved, 8 were full length, and further analysis revealed that they all belong to the XoxF5-type PQQ-MDH (Keltjens et al. 2014). We failed to detect the traditional MxaFI-type methanol dehydrogenases in our genome MAGs or any unbinned contig/scaffold. Consistent with our findings, the lack of *mxaFI* genes has been reported in methylotrophic microorganisms found in various habitats (Beck et al. 2014; Keltjens et al. 2014; Mustakhimov et al. 2013; Padilla et al. 2017; Ramachandran and Walsh 2015). The lower abundance of genes encoding proteins involved in methanotrophy could reflect the shift towards lower CH_4_ concentrations at the sampling site observed from 2003 and 2017 by previous researchers (Amend et al. 2003; Fagorzi et al. 2019).

Consistent with the oxic nature of the samples, our metagenome does not show much potential for methanogenic reactions, methanogens are probably present in the deeper anaerobic layers. In Pantelleria soil, *Methanocella conradii* related sequences were found to dominate the soil layer below 15 cm (Picone et al. 2020). We detected only 1 gene copy of formylmethanofuran dehydrogenase that catalyzes the first step in the reduction of CO_2_ in methanogenic Archaea.

### Carbon assimilation and CO oxidation

Most (autotrophic) members of Proteobacteria phyla fix carbon via ribulose-1,5-bisphosphate carboxylase/oxygenase (RuBisCO) or use organic compounds, like formaldehyde to start assimilation. Microorganisms possess different formaldehyde-reduction/oxidation systems: (i) formaldehyde incorporation via the ribulose monophosphate pathway (RuMP); (ii) formaldehyde assimilation via the serine pathway; (iii) formaldehyde oxidation by dye-linked formaldehyde dehydrogenase; (iv) tetrahydrofolate (H_4_F)-dependent formaldehyde oxidation; (v) tetrahydromethanopterin (H_4_MPT)-dependent formaldehyde oxidation; (vi) thiol (glutathione/mycothiol)—dependent formaldehyde oxidation (Chistoserdova 2011; Vorholt 2002).

In Vulcano soil, 7.5% of the genes identified in carbon metabolism catalyze the conversion of formaldehyde into CO_2_ which yields NAD(P)H. The abundance of *frmAB* genes suggests that it mostly happens via glutathione-dependent oxidation. Moreover, 3.8% of genes incorporate formaldehyde as organic carbon via the RuMP and serine pathways (Fig. 2). Formaldehyde is generated from the methylotrophic metabolism; in our metagenome, this lifestyle (a unique ability of microorganisms to live on C1 molecules) is well represented.

The CO_2_ could become organic carbon through RuBisCO (Prywes et al. 2023) and the oxygen-tolerant Calvin-Benson-Bassham (CBB) cycle, which accounts for a small part (1.8%) of the total amount of genes involved in carbon metabolism. Otherwise, it can be converted into acetyl-CoA through the Wood-Ljungdahl pathway or the reductive tricarboxylic acid (rTCA) cycle represented by 3.4% and 14% of carbon metabolism genes, respectively (Fig. 2).

The abundance of the Wood-Ljungdahl pathway encoded by the carbon monoxide dehydrogenase/acetyl-CoA synthase is quite unusual, given the aerobic nature of the sampling site. It is commonly found in obligate anaerobes in a broad range of phylogenetic classes, diverse acetogens, methanogenic Archaea, sulfate reducers and Planctomycetes that carry out anammox reaction (Ragsdale and Pierce 2008).

**Fig. 3:**
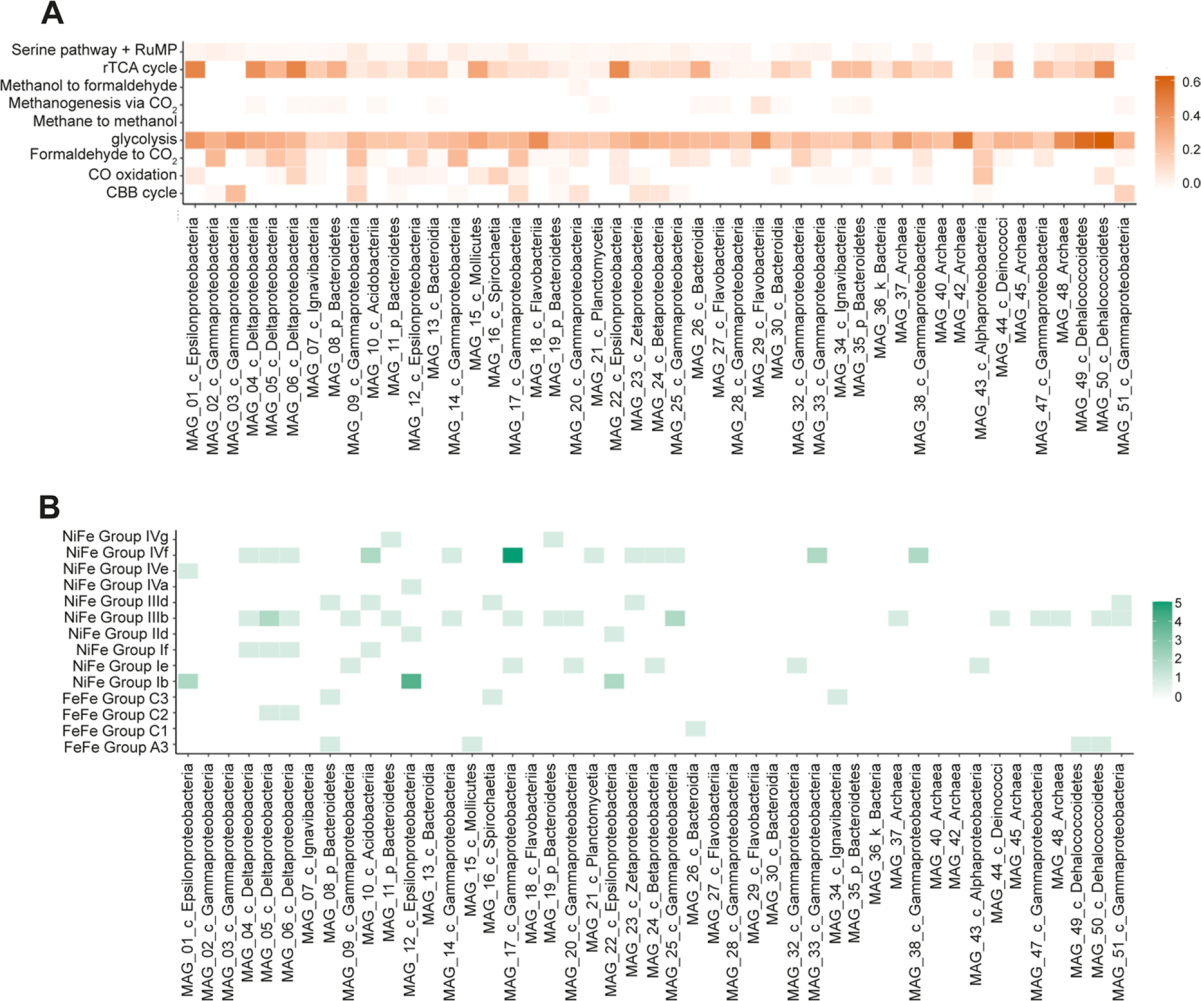
Relative abundance of genes in the most complete MAGs assembled from Vulcano metagenome. **A** Proportion of genes involved in the carbon cycle related to the total amount of genes of the entire MAG. **B** The number of copies of hydrogenase genes

The energy-efficient yet oxygen-sensitive rTCA cycle, known to be the critical pathway on which phototrophic organisms rely, predominates the carbon fixation strategy in our metagenome. Only recently has its biological niche been broadening to non-phototrophic autotrophs as well. For example, the hyperthermophilic chemolithotrophs *Thermoproteus*, *Sulfolobus*, and *Aquifex* use the rTCA cycle, as do certain mesophilic sulfur chemolithotrophic Bacteria such as *Nitrospira* and *Sulfurimonas*, of which the latter is highly represented in our metagenome (Fig. 3A). Interestingly, three-quarters of genes involved in the rTCA cycle were found in unbinned scaffolds and the majority of the encoded proteins showed affiliation to Bacteroidota/Chlorobiota and Desulfobacterales species.

Despite the importance of CO detection in hydrothermal environments, its analysis is often prevented by field and laboratory equipment detection limits (Hogendoorn et al. 2020). Aerobic CO-oxidizers could trigger the production of CO_2_ in Vulcano soil since 4% of genes encode for the heterotrimeric CO dehydrogenases (Fig. 2)—CoxL, a protein containing a [Mo–Cu] cluster, CoxM, a flavoprotein, and CoxS, an iron-sulfur protein. BlastP searches revealed that more than 70% of the Cox-protein showed homology to Gammaproteobacteria, Alphaproteobacteria and Thermodesulfobacteriota.

### Hydrogenase distribution

Hydrogenases are metalloenzymes that belong to three phylogenetically unrelated classes, distinguishable based on the metal content of their H_2_-binding sites: the [NiFe]-, [FeFe]- and [Fe]-hydrogenases (Peters et al. 1998; Shima et al. 2008; Volbeda et al. 1995). In Levante bay, a total of 286 hydrogenase sequences were retrieved, accounting for 3.5% of the total gene number (Fig. 3B). The [Ni–Fe]-hydrogenases are the most widespread, and among them the group 3 [NiFe]-bidirectional and [NiFe] anaerobic uptake hydrogenase were the most abundant (Table 1).

**Table 1:** Distribution of hydrogenases genes in Vulcano metagenome

Type of hydrogenase^a^	No. of genes	Relative abundance of genes (%)
[NiFe] Aerobic uptake	1	0.28
[NiFe] Anaerobic uptake	75	26
[NiFe] Bidirectional	98	34
[NiFe] Evolving	54	18.8
[FeFe] Evolving	23	8
[NiFe] Regulatory	7	2.3
[FeFe] Regulatory	28	10

The classification method is based on HyDB and hydrogenases subdivision is adapted from (Greening et al. 2016): [NiFe] aerobic uptake (group 2a), [NiFe] Anaerobic uptake (groups 1b, 1c, 1e, 1f), [NiFe] Bidirectional (groups 3b, 3d), [NiFe] Evolving (groups 4a, 4e, 4f, 4g), [FeFe] Evolving (groups A1, A3, B), [NiFe] Regulatory (2b, 2c, 2d), [FeFe] Regulatory (group C). The subgroups not mentioned (1a, 1d, 1g, 1h, 1i, 1k, 1j, 2e, 3a, 3c, 4b, 4c, 4d, 4h, 4i, A2, A4, and Fe-hydrogenases) were not present in the metagenome

[NiFe] bidirectional hydrogenases (groups 3b, 3d) possess the most extensive distribution in at least 27 bacterial and archaeal phyla. These oxygen-tolerant enzymes are proposed to serve as redox valves that interconvert electrons between NAD(P)H and H_2_ depending on the availability of exogenous electron acceptors. They constitute dominant groups in great lakes, coastal upwelling, and spring ecosystems with moderately acidic pH (Greening et al. 2016; Lindsay et al. 2019).

The [NiFe] anaerobic uptake hydrogenases typical of deep ocean environments were also detected in Vulcano soil. Such enzymes are O_2_ sensitive and mediate anaerobic respiration in strict anaerobes. Even if these findings contrast with the oxic nature of our samples, similar results were found in the metagenomic analysis of Pantelleria soil (Picone et al. 2020). The hydrogen consumption in the verrucomicrobial strains *Methylacidimicrobium tartarophylax* 4AC and *Methylacidimicrobium thermophilum* AP8 has been linked to a type 1b [NiFe] hydrogenase (Mohammadi et al. 2019; Picone et al. 2021). In our metagenome, [NiFe] Group 1b hydrogenases were abundant and phylogenetically linked to the class of Epsilonproteobacteria (Fig. 3B).

Furthermore, the most ancient group 4 [NiFe] evolving hydrogenases showed a relatively high abundance. They are either energy conserving enzymes that ensure the generation of a proton motive force or hydrogenases that recycle the reducing equivalents from fermentative processes through H_2_ production (Vignais and Billoud 2007).

Whereas [NiFe]-hydrogenases are typically involved in H_2_ uptake and oxidation, almost all [FeFe]-hydrogenases catalyze the production of H_2_. Even if in low amounts, these types of hydrogenases were also present in the Levante bay metagenome.

### Nitrogen and sulfur metabolism

**Fig. 4:**
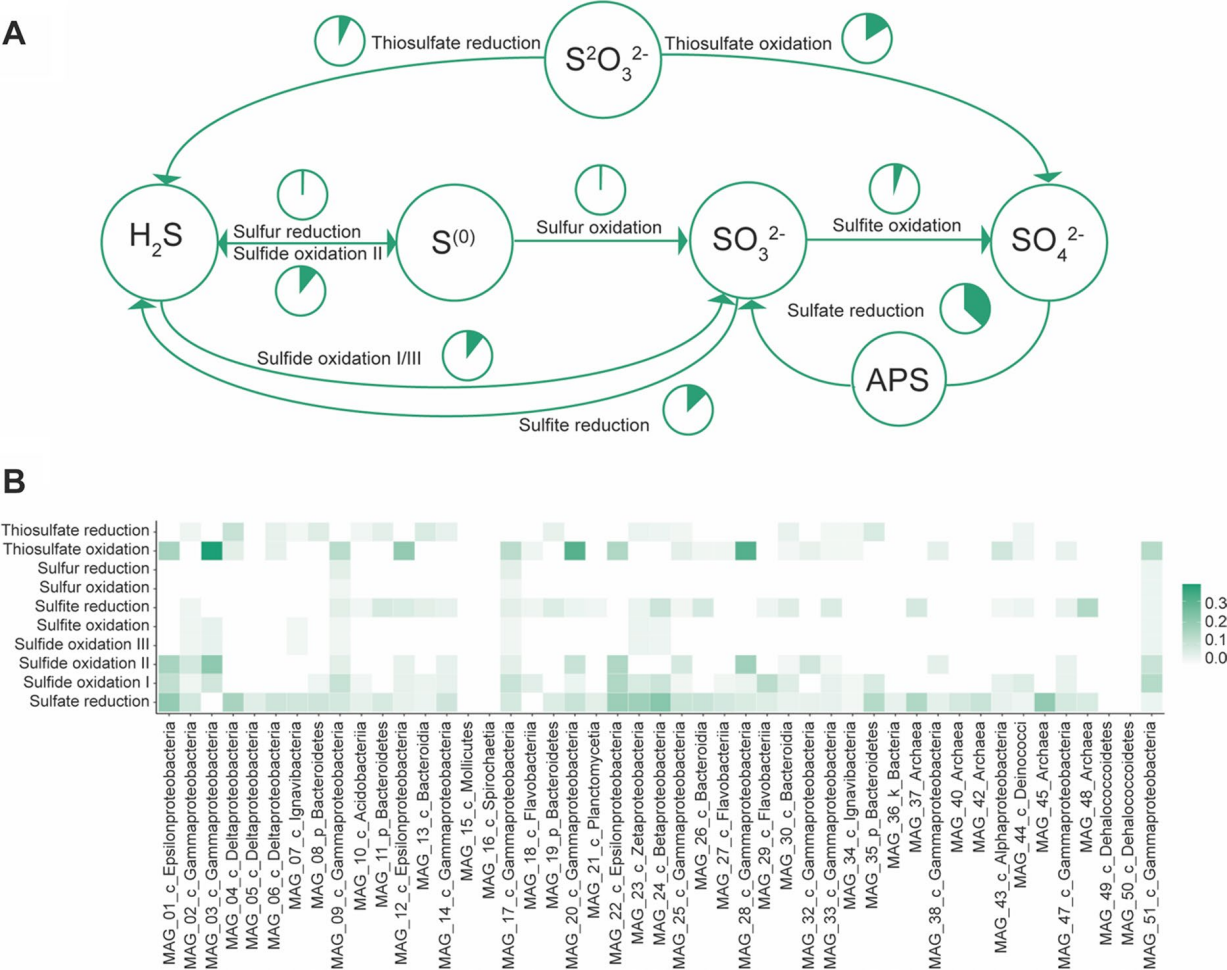
Sulfur cycle overview of Vulcano metagenome. **A** The relative abundance of genes involved in different pathways is calculated as a proportion of the total amount of genes involved in the carbon cycle. **B** The ratio of genes involved in the sulfur cycle relative to the total amount of genes of the entire MAG

One-third of the genes in the Levante bay P7 metagenome accounted for nitrogen and sulfur metabolism. Among them, most of the reads mapped to sulfur cycle genes. We noticed a high abundance of genes involved in assimilatory sulfate reduction. Most of the KEGG enzymes in this category are engaged in sulfidogenesis that may eventually be used to synthesize cysteine/methionine for microbial biomass (Fig. 4A). Sulfate adenyltransferase (*sat/met3, cysN, cysD*), phosphoadenosine phosphosulfate reductase (*cysH*), adenylsulfate kinase (*cysC*), and the CysC/CysN bifunctional enzyme (*cysCN*) are all mapped to assimilatory sulfur processes. With lower abundance, the same sulfate adenyltransferase genes seem to participate in dissimilatory sulfate-reducing reactions. Genes involved in sulfate reduction seem to be present in 41 out of 47 assembled MAGs (Fig. 4B).

The distribution of *soxCD* genes involved in thiosulfate metabolism appears to be mostly confined to members of the Alphaproteobacteria. However, they have been detected in *Thiomicrospira* and Epsilonproteobacteria genomes (Yamamoto et al. 2010). In our metagenome, thiosulfate metabolism tends to shift towards thiosulfate oxidation leading to sulfate generation (Fig. 4A).

Amend, and colleagues reported an elevated amount of sulfate in Levante bay soil (Amend et al. 2003). Moreover, Vulcano is characterized by sulfide-rich areas adjacent to acidified seawater which may be selective for iron- and sulfur-oxidizing bacteria species of *Acidihalobacter* and *Acidithiobacillus* (Simmons and Norris 2002). Genes encoding for sulfur reduction have also been identified in the genome of the type strain of *At. ferrooxidans* (Valdés et al. 2008).

It is worth noting that in three of our MAGs (MAG_09, MAG_17, and MAG_51), it is possible to reconstruct a complete sulfur oxidation pathway (from sulfide to sulfate) two of which—MAG_09 and MAG_17—are 93% similar to the genome of *Acidihalobacter ferrooxidans*.

**Fig. 5:**
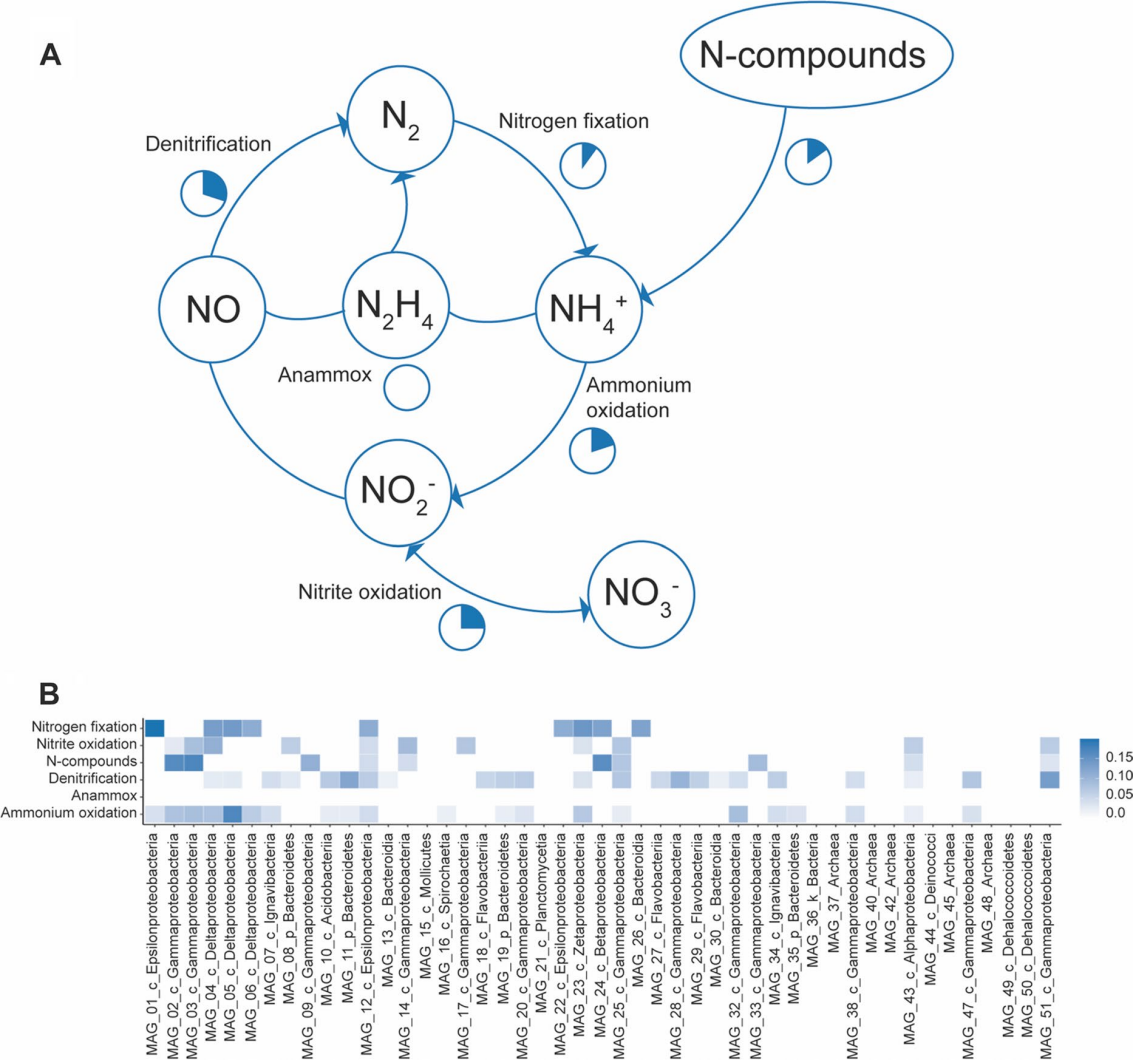
Nitrogen cycle overview of Vulcano metagenome. **A** The relative abundance of genes involved in different pathways is calculated as a proportion of the total amount of genes involved in the carbon cycle. **B** The ratio of genes involved in the nitrogen cycle relative to the total amount of genes of the entire MAG

The nitrogen cycle in Levante bay is limited to some low abundance genes. Denitrification and nitrite oxidation/nitrate reduction accounted for the higher number of genes (Fig. 5A). The dissimilatory mechanism through nitrate reductase seems preferred over the assimilatory one. In denitrification, subunits B and C of nitric oxide reductase (*norB, norC)* are more abundant than other subunits performing denitrification and cause nitrogen to be emitted from the soil. The MAG with the most complete set of genes involved in the nitrogen cycle is MAG_12, 100% similar to *Arcobacter* sp. F2176, a Gram-negative and nitrogen-fixing bacterium (Fig. 5B) (Fera et al. 2004).

#### Iron and arsenic metabolism

Ferric iron and sulfate reduction (see paragraph above) together are quantitatively the most crucial terminal electron-accepting processes in both freshwater and anoxic marine environments. As we stated before, Levante bay is home to iron- sulfur-oxidizers, and Fe^2+^ iron concentrations in our samples were reported to be 309 ppm (Amend et al. 2003). For this reason, we mined our assembled metagenome for genes involved in iron transport and regulation using the FeGenie tool (see Materials and Methods).

**Fig. 6:**
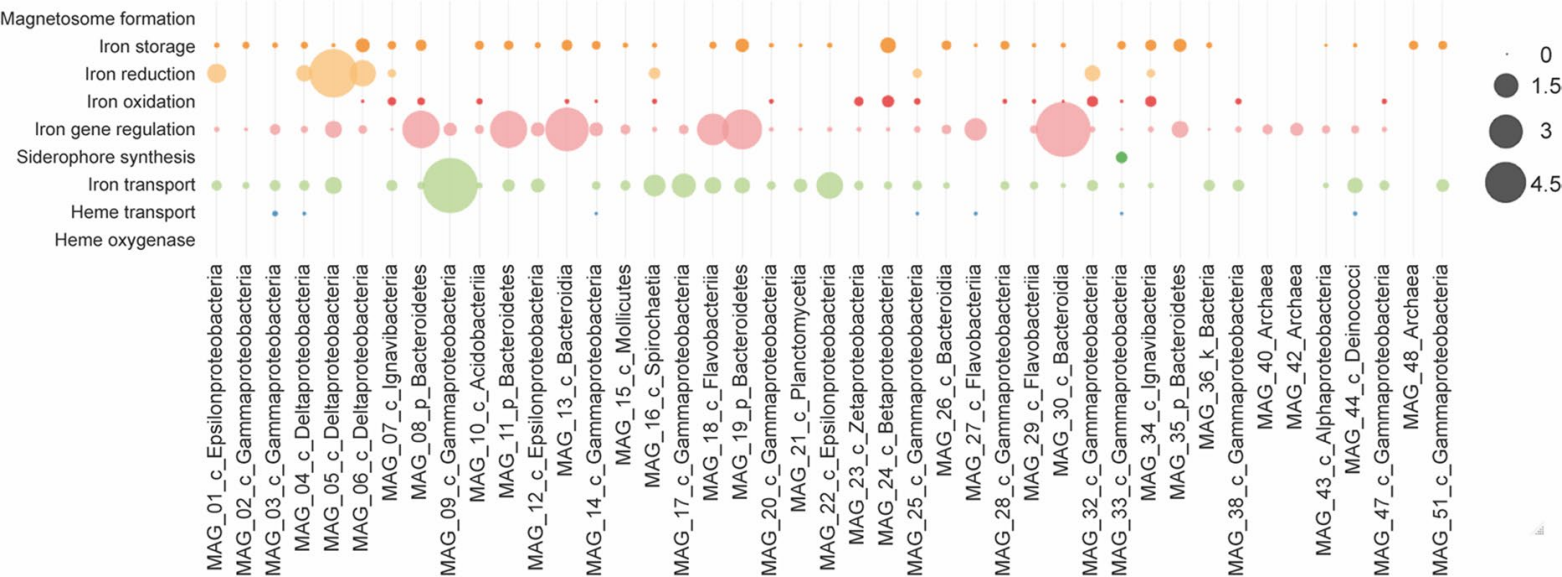
Relative abundance of iron genes calculated using FeGenie tool as a proportion of the total amount of genes involved in iron cycling

We found genes for siderophore synthesis in only one of the MAGs (Fig. 6), while the potential for siderophore transport is found in nearly all of the genomes (data not shown). Among the iron acquisition genes, we detected many genes involved in iron transport with a peak in MAG_09—97.2% similar to genomes of *Acidihalobacter* species—with a gene percentage equal to 4.8%. This is expected, given that iron is a necessary micronutrient for the vast majority of life. Besides, most of the genomes seem to have the capacity to store iron and regulate genes involved in its metabolism; few genomes appear to have the ability to transport heme. In contrast with our expectations, MAG_09 and MAG_17—95.6% similar to *Acidihalobacter* species—seem not to be iron oxidizers. Instead, 19 other MAGs possess genes that can oxidize iron. Moreover, few MAGs show potential for iron reduction; in particular, 4.3% of MAG_05 genes—99,98% similar to *Thiomicrospira kuenenii*—are involved in the iron reduction together with the *Deltaproteobacteria* members represented in the metagenome. We also report iron reduction genes in MAG_01, supposed to represent *Sulfurimonas* species, the most abundant of our metagenome (Fig. 6).

Genome analysis of *At. ferrooxidans* confirmed the presence of an arsenic resistance gene cluster. The cluster includes five genes encoding an arsenate reductase (*arsC*), the arsenate repressor (*arsR*), the divergently-oriented arsenate efflux pump (*arsB*), and a hypothetical protein (*arsH*) (Valdés et al. 2008). To check for the presence of arsenic detoxification or metabolism genes in Vulcano soil, we analyzed the presence of the arsenate reductase (*arsC*) and the two subunits of the arsenite oxidase (*aoxAB*). While none of the genomes show potential to oxidize arsenite, 80% of the MAGs possess 1 up to 5—found in MAG_09—genes that encode for the arsenate reductase.

Researchers separate arsenic-related genes into two categories: resistance and metabolism. *arsC* is involved in arsenic resistance, or detoxification, meaning that it protects the cell from arsenic but does not detoxify arsenic itself in the environment. Microbial arsenic resistance is reportedly widespread in the environment, and arsenic-resistant organisms have been found in sites with low arsenic concentrations. While the number of identified microorganisms with arsenic resistance genes continues to grow, the full scope of arsenic detoxification and metabolism gene distribution is unknown (Dunivin et al. 2019).

## Conclusions

We covered in this study the microbial diversity and metabolic potential of Levante bay on Vulcano island. The soil is populated by representatives of the genera Proteobacteria, Bacteroidetes, Epsilonbacteraeota, and Firmicutes*.* The carbon fixation pathways were mainly represented by the rTCA cycle, followed by the Serine and RuMP pathways. A pMMO operon, a high number of genes involved in methanol, formaldehyde and formate oxidation were retrieved from the metagenome. The study showed as well the potential of CO oxidation and sulfur metabolism. The sulfide/sulfur-oxidizing *Sulfurimonas* is the dominant species in the Levante bay community. This species members use many kinds of reduced sulfur compounds as electron donors and nitrate, nitrite, and oxygen as electron acceptors. Moreover, some of the assembled genomes could be involved in iron metabolism. In total, 47 MAGs, more than 70% complete and less than 10% redundant, have been assembled from the Vulcano metagenome.

## Supplementary Information

Below is the link to the electronic supplementary material.

Supplementary file 1.

## Data Availability

On request raw data supporting the conclusions of this article will be made available by the authors, without any reservation. Sequencing data and MAGs are available at the European Nucleotide Archive (ENA) database under project number PREJB47788.
