# Supplementary material for: Gene-centered metagenome analysis of Vulcano Island soil (Aeolian archipelago, Italy) reveals diverse microbial key players in methane, hydrogen and sulfur cycles
Source: Antonie Van Leeuwenhoek. 2024 Jul 2;117(1):94. doi: 10.1007/s10482-024-01995-5 (PMC11219375; doi:10.1007/s10482-024-01995-5)
Supplement: Supplementary file 1 [file 10482_2024_1995_MOESM1_ESM.pdf]

Antonie van Leeuwenhoek

Supplementary Material

Gene-centered metagenome analysis of Vulcano Island soil (Aeolian archipelago, Italy) reveals diverse microbial key players in methane, hydrogen and sulfur cycles

Federica Angius[1] Geert Cremers[1], Jeroen Frank[1], Caitlyn Witkowski[2], Arjan Pol[1], Theo A. van Alen[1], Mike S.M. Jetten[1], Huub J.M. Op den Camp[1*] & Tom Berben[1]

[1]Department of Microbiology, Radboud Institute for Biological and Environmental Sciences, Faculty of Science, Radboud University, Nijmegen, the Netherlands

[2] Department of Marine Microbiology and Biogeochemistry, NIOZ, P.O. Box 59, 1790 AB Den Burg, Texel, the Netherlands

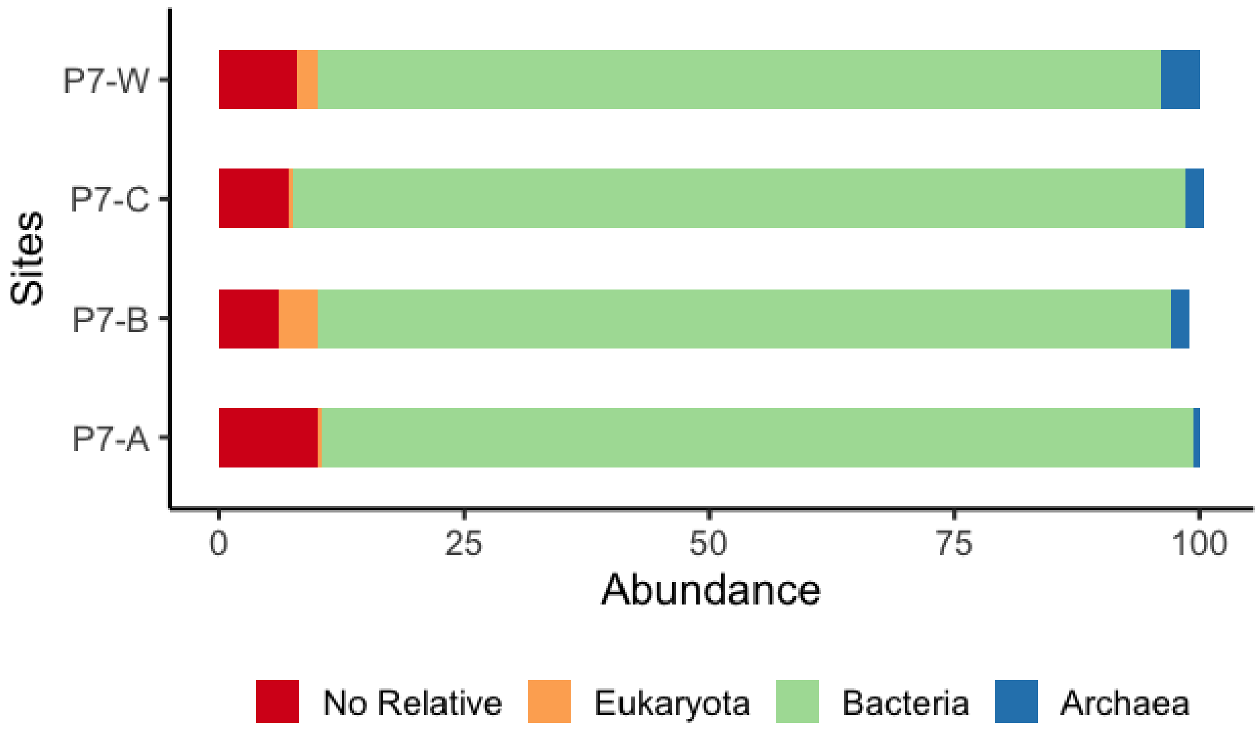

Figure S1. Abundance of representatives from the three domains of life in the Vulcano metagenomes from four different sites..

Table S1. Relative abundance of different DNA extraction at Vulcano metagenome Punto 7 based on 16S rRNA gene sequences. Data represented are at least 1% abundant in one of the different DNA extractions.

	P7-A	P7-B		P7-C		P7-W	
	FD	FD	PS	FD	PEG	FD	PEG
Archaea	0.7	2	2	2	3	4	5
Bacteria	89	87	66	91	91	86	80
Eukaryota	0.4	4	5	0.5	1	2	5
No Relative	10	6	27	7	5	8	10
**Archaea**							
Asgardaeota	0.0	0.0	0.01	0.0	0.0	0.0	0.05
Euryarchaeota	0.4	0.2	0.1	0.1	0.2	0.0	0.0
Nanoarchaeaeota	0.3	1.7	2.0	2.1	2.5	4.2	5.0
**Bacteria**							
Acidobacteria	0.5	0.1	0.1	0.9	1.6	1.3	1.0
Actinobacteria	1.4	0.6	0.9	0.6	0.9	0.3	0.5
Bacteroidetes	8.7	4.7	5.9	17.8	16.5	17.5	15.7
Cyanobacteria	0.2	1.6	0.5	0.5	1.3	1.6	4.0
Deinococcus-Thermus	3.1	3.0	3.9	1.3	1.1	2.6	1.8
Epsilonbacteraeota	0.2	0.1	0.07	28.3	25.4	0.1	0.3
Firmicutes	10.9	0.3	1.4	1.7	1.4	0.6	0.9
Patescibacteria	0.8	1.9	2.3	1.0	1.2	4.4	4.0
Planctomycetes	0.2	0.6	0.5	1.0	2.2	2.7	4.0
Proteobacteria	62.1	73.7	48.6	29.5	30.7	47.3	41.8
Spirochaetes	0.2	0.4	0.8	1.1	2.5	1.3	1.1
Tenericutes	0.04	0.03	0.05	3.1	1.9	1.0	1.0
Thermotogae	0.0	0.0	0.0	1.5	0.9	0.03	0.05
Verrucomicrobia	0.3	0.4	0.2	0.8	1.1	2.9	1.8
Other	10.7	10.7	32.67	8.7	8.6	12.17	17
**Proteobacteria**							
9M32	5.2	4.9	4.6	2.2	2.7	5.5	2.2
Acidihalobacter	16.9	1.8	0.8	0.8	0.6	0.3	0.4
Alcanivorax	1.6	2.4	1.1	0.0	0.1	0.1	0.4
Defluviimonas	0.3	3.3	1.6	0.1	0.1	0.3	0.2
Desulfovibrio	0.0	0.0	0.0	1.1	0.6	0.1	0.0
Desulfuromusa	1.1	0.3	0.1	1.1	0.7	0.6	0.7
Geothermobacter	1.9	0.4	0.2	0.1	0.1	0.8	0.6
Halothiobacillus	0.0	1.7	0.2	0.1	0.01	0.2	0.2
Luteibacter	0.6	2.4	1.1	0.2	0.3	0.8	0.5
Malomonas	0.02	0.0	0.0	1.3	0.0	0.8	0.0
Marinobacter	2.2	12.3	5.3	0.6	1.0	1.1	2.0
Mariprofundus	0.04	0.3	0.05	1.6	1.2	1.8	1.5
Methylonatrum	1.2	0.2	0.2	0.0	0.1	0.1	0.03
Methylophaga	0.1	1.2	0.9	0.3	0.3	0.3	0.3
Mizugakiibacter	0.3	1.2	0.1	0.04	0.1	0.1	0.1
Oleiagrimonas	4.2	11.0	3.0	0.6	0.7	2.2	1.5
OM182 clade	0.3	0.7	0.5	0.04	0.1	1.2	0.8
Salinisphaera	1.0	4.2	8.4	0.04	0.0	0.1	0.2
Spiribacter	1.2	0.6	0.4	0.02	0.1	0.1	0.1
Thiomicrospira	0.0	0.05	0.0	3.0	3.5	0.01	0.03
Uncultured	4.7	6.7	5.0	2.9	3.5	7.2	6.4
**Epsilonbacteraeota**							
Arcobacter	0.0	0.0	0.0	3.3	3.1	0.0	0.02

Sulfurimonas	0.3	0.1	0.1	25.0	22.3	0.1	0.2
**Bacteroidetes**							
Muricauda	0.0	0.0	0.0	1.9	1.4	1.8	1.4
Prolixibacter	0.6	0.0	0.0	3.3	1.8	0.7	0.3
VC2.1 Bac22	4.6	0.4	0.1	2.7	2.4	1.5	0.8
Yeosuana	0.3	0.05	0.02	0.8	0.9	1.3	1.0
Uncultured	0.4	1.2	1.6	2.3	2.3	2.4	2.8
**Firmicutes**							
Desulfosporosinus	9.8	0.0	0.02	0.04	0.04	0.0	0.1

Table S2. Taxonomy, completeness and redundancy of assembled MAGs.

MAG number	Taxonomy	Completeness	Redundancy
MAG_01	k__Bacteria;p__Proteobacteria;c__Epsilonproteobacteria;o__Campylobacterales;f__Helicobacteraceae; g__Sulfurimonas	99,59	2,66
MAG_02	k__Bacteria;p__Proteobacteria;c__Gammaproteobacteria; o__Oceanospirillales	99,57	0,07
MAG_03	k__Bacteria;p__Proteobacteria;c__Gammaproteobacteria;o__Thiotrichales;f__Piscirickettsiaceae;g__Thiomicrospira	99,39	0
MAG_04	k__Bacteria;p__Proteobacteria;c__Deltaproteobacteria;o__Desulfuromonadales;f__Geobacteraceae	99,35	0,65
MAG_05	k__Bacteria;p__Proteobacteria;c__Deltaproteobacteria;o__Desulfuromonadales;f__Geobacteraceae	99,35	0,65
MAG_06	k__Bacteria;p__Proteobacteria;c__Deltaproteobacteria;o__Desulfuromonadales;f__Geobacteraceae	98,71	0,48
MAG_07	k__Bacteria;p__Ignavibacteriae;c__Ignavibacteria;o__Ignavibacteriales	98,6	1,12
MAG_08	k__Bacteria;p__Bacteroidetes	97,72	0,57
MAG_09	k__Bacteria;p__Proteobacteria;c__Gammaproteobacteria	97,59	3
MAG_10	k__Bacteria;p__Acidobacteria;c__Acidobacteriia;o__Acidobacteriales;f__Acidobacteriaceae;g__Acidobacterium	97,44	1,07
MAG_11	k__Bacteria;p__Bacteroidetes	97,27	3,39
MAG_12	k__Bacteria;p__Proteobacteria;c__Epsilonproteobacteria;o__Campylobacterales;f__Campylobacteraceae; g__Arcobacter	97,15	6,44
MAG_13	k__Bacteria;p__Bacteroidetes;c__Bacteroidia;o__Bacteroidales;f__Porphyromonadaceae	97,1	5,44
MAG_14	k__Bacteria;p__Proteobacteria;c__Gammaproteobacteria;o__Oceanospirillales	96,97	4,07
MAG_15	k__Bacteria;p__Tenericutes;c__Mollicutes	96,67	8,67
MAG_16	k__Bacteria;p__Spirochaetes;c__Spirochaetia;o__Spirochaetales;f__Spirochaetaceae	96,48	8,73
MAG_17	k__Bacteria;p__Proteobacteria;c__Gammaproteobacteria	96,12	7,38
MAG_18	k__Bacteria;p__Bacteroidetes;c__Flavobacteriia; o__Flavobacteriales;f__Flavobacteriaceae	95,95	6,86
MAG_19	k__Bacteria;p__Bacteroidetes	95,08	3,22
MAG_20	k__Bacteria;p__Proteobacteria;c__Gammaproteobacteria;o__Xanthomonadales;f__Xanthomonadaceae	95,02	0,86
MAG_21	k__Bacteria;p__Planctomycetes;c__Planctomycetia;o__Planctomycetales;f__Planctomycetaceae	94,32	1,14
MAG_22	k__Bacteria;p__Proteobacteria;c__Epsilonproteobacteria;o__Campylobacterales;f__Helicobacteraceae;g__Sulfurimonas	94,2	7,17
MAG_23	k__Bacteria;p__Proteobacteria;c__Zetaproteobacteria;o__Mariprofundales;f__Mariprofundaceae;g__Mariprofundus;s__Mariprofundus_ferrooxydans	94,07	2,54
MAG_24	k__Bacteria;p__Proteobacteria;c__Betaproteobacteria	93,42	1,6
MAG_25	k__Bacteria;p__Proteobacteria;c__Gammaproteobacteria;o__Oceanospirillales	93,05	1,86
MAG_26	k__Bacteria;p__Bacteroidetes;c__Bacteroidia;o__Bacteroidales;f__Porphyromonadaceae	92,92	2,2
MAG_27	k__Bacteria;p__Bacteroidetes;c__Flavobacteriia;o__Flavobacteriales	92,72	0,56

MAG_28	k__Bacteria;p__Proteobacteria;c__Gammaproteobacteria;o__Xanthomonadales;f__Xanthomonadaceae	92,7	1,75
MAG_29	k__Bacteria;p__Bacteroidetes;c__Flavobacteriia;o__Flavobacteriales;f__Flavobacteriaceae	90,17	7,19
MAG_30	k__Bacteria;p__Bacteroidetes;c__Bacteroidia;o__Bacteroidales;f__Porphyromonadaceae	88,25	5,65
MAG_32	k__Bacteria;p__Proteobacteria;c__Gammaproteobacteria	87,18	1,56
MAG_33	k__Bacteria;p__Proteobacteria;c__Gammaproteobacteria;o__Xanthomonadales	86,92	7,19
MAG_34	k__Bacteria;p__Ignavibacteriae;c__Ignavibacteria;o__Ignavibacteriales	84,95	4,82
MAG_35	k__Bacteria;p__Bacteroidetes	84,1	2,69
MAG_36	k__Bacteria	83,79	5,65
MAG_37	k__Archaea (root)	83,64	0,93
MAG_38	k__Bacteria;p__Proteobacteria;c__Gammaproteobacteria	82,44	0,97
MAG_40	k__Archaea (root)	81,31	0
MAG_42	k__Archaea (root)	80,84	1,4
MAG_43	k__Bacteria;p__Proteobacteria;c__Alphaproteobacteria;o__Rhodobacterales;f__Rhodobacteraceae	80,74	5,83
MAG_44	k__Bacteria;p__Deinococcus-Thermus;c__Deinococci; o__Deinococcales;f__Deinococcaceae	80,08	4,4
MAG_45	k__Archaea (root)	78,89	0,93
MAG_47	k__Bacteria;p__Proteobacteria;c__Gammaproteobacteria	77,31	1,38
MAG_48	k__Archaea (root)	74,53	0
MAG_49	k__Bacteria;p__Chloroflexi;c__Dehalococcoidetes	73,43	1,98
MAG_50	k__Bacteria;p__Chloroflexi;c__Dehalococcoidetes	71,29	0
MAG_51	k__Bacteria;p__Proteobacteria;c__Gammaproteobacteria	70,8	3,12

Table S3. List of HMM marker genes used for metagenomic analysis

sulfide dehydrogenase [flavocytochrome c] flavoprotein chain (EC:1.8.2.3)
cytochrome subunit of sulfide dehydrogenase
sulfide:quinone oxidoreductase [EC:1.8.5.4]
dissimilatory sulfite reductase alpha subunit [EC:1.8.99.5]
dissimilatory sulfite reductase beta subunit [EC:1.8.99.5]
anaerobic sulfite reductase subunit A
anaerobic sulfite reductase subunit B
anaerobic sulfite reductase subunit C
sulfur oxygenase/reductase [EC:1.13.11.55]
sulfur dioxygenase [EC:1.13.11.18]
sulfur-oxidizing protein SoxA; L-cysteine S-thiosulfotransferase [EC:2.8.5.2]
sulfur-oxidizing protein SoxB; S-sulfosulfanyl-L-cysteine sulfohydrolase [EC:3.1.6.20]
sulfane dehydrogenase subunit SoxC;
sulfur-oxidizing protein SoxY
S-disulfanyl-L-cysteine oxidoreductase SoxD [EC:1.8.2.6]
L-cysteine S-thiosulfotransferase [EC:2.8.5.2]
sulfur-oxidizing protein SoxZ
adenylylsulfate reductase, subunit A [EC:1.8.99.2]
adenylylsulfate reductase, subunit B [EC:1.8.99.2]
sulfate adenylyltransferase [EC:2.7.7.4]
sulfate adenylyltransferase subunit 2 [EC:2.7.7.4]
3'-phosphoadenosine 5'-phosphosulfate synthase [EC:2.7.7.4 2.7.1.25]
adenylylsulfate kinase [EC:2.7.1.25]
sulfate adenylyltransferase subunit 1 [EC:2.7.7.4]
bifunctional enzyme CysN/CysC [EC:2.7.7.4 2.7.1.25]
phosphoadenosine phosphosulfate reductase [EC:1.8.4.8 1.8.4.10]
sulfite reductase (NADPH) flavoprotein alpha-component [EC:1.8.1.2]
sulfite reductase (NADPH) hemoprotein beta-component [EC:1.8.1.2]
sulfite reductase (ferredoxin) [EC:1.8.7.1]
sulfur reductase molybdopterin subunit
sulfur reductase FeS subunit
sulfur reductase membrane anchor
thiosulfate reductase / polysulfide reductase chain A [EC:1.8.5.5]
thiosulfate reductase electron transport protein
thiosulfate reductase cytochrome b subunit
sulfite dehydrogenase (cytochrome C) [EC:1.8.2.1]
sulfite dehydrogenase (cytochrome C) subunit B
sulfite oxidase
sulfite dehydrogenase (quinone) subunit SoeA
sulfite dehydrogenase (quinone) subunit SoeB
sulfite dehydrogenase (quinone) subunit SoeC
Fe-Fe hydrogenase Group A1
Fe-Fe hydrogenase Group A2
Fe-Fe hydrogenase Group A3
Fe-Fe hydrogenase Group A4
Fe-Fe hydrogenase Group B
Fe-Fe hydrogenase Group C1
Fe-Fe hydrogenase Group C2
Fe-Fe hydrogenase Group C3
Fe-Fe hydrogenase Group Fe
Ni-Fe hydrogenase Group 1a
Ni-Fe hydrogenase Group Ib
Ni-Fe hydrogenase Group Ic
Ni-Fe hydrogenase Group Id
Ni-Fe hydrogenase Group Ie
Ni-Fe hydrogenase Group If
Ni-Fe hydrogenase Group Ig
Ni-Fe hydrogenase Group Ih
Ni-Fe hydrogenase Group Ii
Ni-Fe hydrogenase Group Ij
Ni-Fe hydrogenase Group Ik

Ni-Fe hydrogenase Group IIa
Ni-Fe hydrogenase Group IIb
Ni-Fe hydrogenase Group IIc
Ni-Fe hydrogenase Group IId
Ni-Fe hydrogenase Group IIe
Ni-Fe hydrogenase Group IIIa
Ni-Fe hydrogenase Group IIIb
Ni-Fe hydrogenase Group IIIc
Ni-Fe hydrogenase Group IIId
Ni-Fe hydrogenase Group IVa
Ni-Fe hydrogenase Group IVb
Ni-Fe hydrogenase Group IVc
Ni-Fe hydrogenase Group IVd
Ni-Fe hydrogenase Group IVe
Ni-Fe hydrogenase Group IVf
Ni-Fe hydrogenase Group IVg
Ni-Fe hydrogenase Group IVh
Ni-Fe hydrogenase Group IVi
methane/ammonia monooxygenase subunit A [EC:1.14.18.3 1.14.99.39]
methane/ammonia monooxygenase subunit B
methane/ammonia monooxygenase subunit C
methane monooxygenase component A alpha chain
methane monooxygenase component A beta chain
methane monooxygenase component A gamma chain
methane monooxygenase regulatory protein B
methane monooxygenase component D
methyl-coenzyme M reductase alpha subunit [EC:2.8.4.1]
methyl-coenzyme M reductase beta subunit [EC:2.8.4.1]
methyl-coenzyme M reductase gamma subunit [EC:2.8.4.1]
methyl-coenzyme M reductase subunit C
methane/ammonia monooxygenase subunit A [EC:1.14.18.3 1.14.99.39]
nitrogenase iron-iron protein, alpha chain [EC:1.18.6.1]
nitrogenase iron-iron protein, beta chain [EC:1.18.6.1]
nitrogenase delta subunit [EC:1.18.6.1]
nitrogenase molybdenum-iron protein alpha chain [EC:1.18.6.1]
nitrogenase molybdenum-iron protein beta chain [EC:1.18.6.1]
nitrogenase vanadium-iron protein, alpha chain [EC:1.18.6.1]
nitrogenase vanadium-iron protein beta chain [EC:1.18.6.1]
nitrogenase vanadium-iron protein delta chain [EC:1.18.6.1]
nitrogenase iron protein NifH [EC:1.18.6.1]
nitrate reductase / nitrite oxidoreductase, alpha subunit [EC:1.7.5.1 1.7.99.-]
nitrate reductase / nitrite oxidoreductase, beta subunit [EC:1.7.5.1 1.7.99.-]
periplasmic nitrate reductase NapA [EC:1.7.99.-]
nitrate reductase cytochrome c-type subunit
nitrate reductase / nitrite oxidoreductase, alpha subunit [EC:1.7.5.1 1.7.99.-]
nitrate reductase / nitrite oxidoreductase, beta subunit [EC:1.7.5.1 1.7.99.-]
nitrate reductase gamma subunit
nitrate reductase (NAD(P)H) [EC:1.7.1.1 1.7.1.2 1.7.1.3]
ferredoxin-nitrate reductase [EC:1.7.7.2]
assimilatory nitrate reductase catalytic subunit [EC:1.7.99.-]
assimilatory nitrate reductase electron transfer subunit [EC:1.7.99.-]
cytochrome c nitrite reductase small subunit
ferredoxin-nitrite reductase [EC:1.7.7.1]
nitrite reductase (cytochrome c-552) [EC:1.7.2.2]
nitrite reductase (NAD(P)H) [EC:1.7.1.4]
cytochrome c nitrite reductase, NrfD subunit [EC:1.7.2.2]
nitrite reductase (NADH) large subunit [EC:1.7.1.15]
nitrite reductase (NADH) small subunit [EC:1.7.1.15]
nitrite reductase (NO-forming) [EC:1.7.2.1]
nitrite reductase (NO-forming) / hydroxylamine reductase [EC:1.7.2.1 1.7.99.1]
octaheme c-type cytochrome, tetrathionate reductase family [EC:1.7.2.2]
nitric oxide reductase subunit B [EC:1.7.2.5]
nitric oxide reductase subunit C

nitrous-oxide reductase [EC:1.7.2.4]
hydrazine oxidoreductase A hydroxylamine oxidoreductase hydrazine dehydrogenase [EC:1.7.2.8]
hydrazine synthase subunit A [EC 1.7.2.7]
hydrazine synthase subunit B [EC 1.7.2.7]
hydrazine synthase subunit C [EC:1.7.2.7]
hydroxylamine dehydrogenase [EC:1.7.2.6]
urease subunit alpha [EC:3.5.1.5]
urease subunit beta [EC:3.5.1.5]
urease subunit gamma [EC:3.5.1.5]
urease subunit beta/gamma [EC:3.5.1.5]
urease [EC:3.5.1.5]
cyanate lyase [EC:4.2.1.104]
nitrile hydratase subunit alpha [EC:4.2.1.84]
nitrile hydratase subunit beta [EC:4.2.1.84]
cytochrome c oxidase subunit I [EC:1.9.3.1]
cytochrome c oxidase subunit I,coxAC; cytochrome c oxidase subunit I+III
cytochrome c oxidase subunit II [EC:1.9.3.1]
cytochrome c oxidase cbb3-type subunit I [EC:1.9.3.1]
cytochrome c oxidase cbb3-type subunit II
cytochrome c oxidase cbb3-type subunit III
cytochrome o ubiquinol oxidase subunit II [EC:1.10.3.10]
cytochrome o ubiquinol oxidase subunit IV
heme o synthase [EC:2.5.1.141]
cytochrome bd ubiquinol oxidase subunit I [EC:1.10.3.14]
cytochrome bd ubiquinol oxidase subunit II [EC:1.10.3.14]
cytochrome aa3-600 menaquinol oxidase subunit II [EC:1.10.3.12]
cytochrome aa3-600 menaquinol oxidase subunit I [EC:1.10.3.12]
cytochrome aa3-600 menaquinol oxidase subunit III,qoxC
PPQ-depenent methanol dehydrogenase [EC:1.1.2.7]
PPQ-depenent methanol dehydrogenase [EC:1.1.2.7]
methanol dehydrogenase (cytochrome c) subunit 2
methanol dehydrogenase [EC:1.1.1.244]
NDMA-dependent methanol dehydrogenase EC 1.1.1.244
alcohol oxidase [EC:1.1.3.13]
methylamine dehydrogenase light chain [EC:1.4.9.1]
methylamine dehydrogenase heavy chain [EC:1.4.9.1]
[methyl-Co(III) methylamine-specific corrinoid protein]:coenzyme M methyltransferase [EC:2.1.1.247]
[methyl-Co(III) methanol-specific corrinoid protein]:coenzyme M methyltransferase [ec:2.1.1.246]
glutathione-independent formaldehyde dehydrogenase [EC:1.2.1.46]
S-formylglutathione hydrolase [EC:3.1.2.12]
S-(hydroxymethyl)glutathione dehydrogenase / alcohol dehydrogenase [EC:1.1.1.284 1.1.1.1]
S-(hydroxymethyl)mycothiol dehydrogenase [EC:1.1.1.306]
5,6,7,8-tetrahydromethanopterin hydro-lyase [EC:4.2.1.147]
formylmethanofuran--tetrahydromethanopterin N-formyltransferase [EC:2.3.1.101]
methenyltetrahydromethanopterin cyclohydrolase [EC:3.5.4.27]
formate dehydrogenase [EC:1.17.1.9]
formate dehydrogenase major subunit [EC:1.17.1.9]
formate dehydrogenase beta subunit [EC:1.17.1.9]
formate dehydrogenase iron-sulfur subunit
formate dehydrogenase subunit gamma
formate dehydrogenase subunit delta [EC:1.17.1.9]
formate dehydrogenase (coenzyme F420) beta subunit [EC:1.17.98.3 1.8.98.6]
formate dehydrogenase (coenzyme F420) alpha subunit [EC:1.17.98.3 1.8.98.6]
formate dehydrogenase (NADP+) alpha subunit [EC:1.17.1.10]
formate dehydrogenase (NADP+) beta subunit [EC:1.17.1.10]
aerobic carbon-monoxide dehydrogenase small subunit [EC:1.2.5.3]
aerobic carbon-monoxide dehydrogenase medium subunit [EC:1.2.5.3]
aerobic carbon-monoxide dehydrogenase large subunit [EC:1.2.5.3]
ribulose-bisphosphate carboxylase large chain [EC:4.1.1.39] Form I
ribulose-bisphosphate carboxylase large chain [EC:4.1.1.39] Form II
ribulose-bisphosphate carboxylase large chain [EC:4.1.1.39] Form II/III
ribulose-bisphosphate carboxylase large chain [EC:4.1.1.39] Form III
ribulose-bisphosphate carboxylase large chain [EC:4.1.1.39] Form IV

ribulose-bisphosphate carboxylase small chain [EC:4.1.1.39]
phosphoribulokinase [EC:2.7.1.19]
acetyl-CoA synthase [EC:2.3.1.169]
acetyl-CoA decarbonylase/synthase complex subunit beta [EC:2.3.1.-]
acetyl-CoA decarbonylase/synthase complex subunit alpha [EC:1.2.7.4]
acetyl-CoA decarbonylase/synthase complex subunit gamma [EC:2.1.1.245]
acetyl-CoA decarbonylase/synthase complex subunit epsilon
acetyl-CoA decarbonylase/synthase complex subunit delta [EC:2.1.1.245]
anaerobic carbon-monoxide dehydrogenase catalytic subunit [EC:1.2.7.4]
anaerobic carbon-monoxide dehydrogenase iron sulfur subunit
glycine hydroxymethyltransferase [EC:2.1.2.1]
malate-CoA ligase subunit beta [EC:6.2.1.9]
malate-CoA ligase subunit alpha [EC:6.2.1.9]
3-hexulose-6-phosphate synthase [EC:4.1.2.43]
bifunctional enzyme Fae/Hps [EC:4.2.1.147 4.1.2.43]
3-hexulose-6-phosphate synthase / 6-phospho-3-hexuloisomerase [EC:4.1.2.43 5.3.1.27]
pyruvate ferredoxin oxidoreductase alpha subunit [EC:1.2.7.1]
pyruvate ferredoxin oxidoreductase beta subunit [EC:1.2.7.1]
pyruvate ferredoxin oxidoreductase delta subunit [EC:1.2.7.1]
pyruvate ferredoxin oxidoreductase gamma subunit [EC:1.2.7.1]
pyruvate-ferredoxin/flavodoxin oxidoreductase [EC:1.2.7.1 1.2.7.-]
malyl-CoA/(S)-citramalyl-CoA lyase [ec:4.1.3.24,4.1.3.25]
succinate semialdehyde reductase (NADPH) [EC:1.1.1.-]
2-oxoglutarate/2-oxoacid ferredoxin oxidoreductase subunit alpha [EC:1.2.7.3 1.2.7.11]
2-oxoglutarate/2-oxoacid ferredoxin oxidoreductase subunit beta [EC:1.2.7.3 1.2.7.11]
2-oxoglutarate ferredoxin oxidoreductase subunit delta [EC:1.2.7.3]
2-oxoglutarate ferredoxin oxidoreductase subunit gamma [EC:1.2.7.3]
ATP-citrate lyase alpha-subunit [EC:2.3.3.8]
ATP-citrate lyase beta-subunit [EC:2.3.3.8]
2-haloacid dehalogenase [EC:3.8.1.2]
tetrachloroethene reductive dehalogenase catalytic subunit PceA [EC:1.21.99.5]
DMSO reductase family type II enzyme, molybdopterin subunit / Perchlorate reductase subunit alpha (EC:1.97.1.-)
selenate/chlorate reductase subunit alpha ; clrA, serA [EC:1.97.1.9 1.97.1.1]
DMSO reductase family type II enzyme, iron-sulfur subunit / Perchlorate reductase subunit beta
clrb, serB; selenate/chlorate reductase subunit beta [EC:1.97.1.9 1.97.1.1]
serC, clrC; selenate/chlorate reductase subunit gamma [ec:1.97.1.9,1.97.1.1]
chlorite dismutase [EC:1.13.11.49]
arsenite oxidase small subunit [EC:1.20.2.1 1.20.9.1]
arsenite oxidase large subunit [EC:1.20.2.1 1.20.9.1]
arsenate reductase (glutaredoxin) [EC:1.20.4.1]
arsenate reductase (thioredoxin) [EC.1.20.4.- 3.1.3.48]
arsenate reductase, glutathione/glutaredoxin type
putative selenate reductase FAD-binding subunit
probable selenate reductase, molybdenum-binding subunit
putative selenate reductase [EC:1.97.1.9]
decaheme c-type cytochrome, OmcA/MtrC family
decaheme-associated outer membrane protein, MtrB/PioB family
